# Restoration of Central Programmed Movement Pattern by Temporal Electrical Stimulation-Assisted Training in Patients with Spinal Cerebellar Atrophy

**DOI:** 10.1155/2015/462182

**Published:** 2015-08-31

**Authors:** Ying-Zu Huang, Yao-Shun Chang, Miao-Ju Hsu, Alice M. K. Wong, Ya-Ju Chang

**Affiliations:** ^1^Department of Neurology and Neuroscience Research Center, Chang Gung Memorial Hospital, 5 Fusing Street, Kweishan, Taoyuan 333, Taiwan; ^2^School of Medicine, College of Medicine, Chang Gung University, Taoyuan 333, Taiwan; ^3^Department of Physical Therapy and Graduate Institute of Rehabilitation Science, College of Medicine and Healthy Aging Research Center, Chang Gung University, Taoyuan 333, Taiwan; ^4^Department of Physical Medicine and Rehabilitation, Wan Fang Hospital, Taipei Medical University, Taipei 116, Taiwan; ^5^Department of Physical Therapy, College of Health Science, Kaohsiung Medical University, Kaohsiung 807, Taiwan; ^6^Department of Physical Medicine and Rehabilitation, Kaohsiung Medical University Hospital, Kaohsiung 807, Taiwan; ^7^Department of Physical Medicine and Rehabilitation, Chang Gung Memorial Hospital, Taoyuan 333, Taiwan

## Abstract

Disrupted triphasic electromyography (EMG) patterns of agonist and antagonist muscle pairs during fast goal-directed movements have been found in patients with hypermetria. Since peripheral electrical stimulation (ES) and motor training may modulate motor cortical excitability through plasticity mechanisms, we aimed to investigate whether temporal ES-assisted movement training could influence premovement cortical excitability and alleviate hypermetria in patients with spinal cerebellar ataxia (SCA). The EMG of the agonist extensor carpi radialis muscle and antagonist flexor carpi radialis muscle, premovement motor evoked potentials (MEPs) of the flexor carpi radialis muscle, and the constant and variable errors of movements were assessed before and after 4 weeks of ES-assisted fast goal-directed wrist extension training in the training group and of general health education in the control group. After training, the premovement MEPs of the antagonist muscle were facilitated at 50 ms before the onset of movement. In addition, the EMG onset latency of the antagonist muscle shifted earlier and the constant error decreased significantly. In summary, temporal ES-assisted training alleviated hypermetria by restoring antagonist premovement and temporal triphasic EMG patterns in SCA patients. This technique may be applied to treat hypermetria in cerebellar disorders. (This trial is registered with NCT01983670.)

## 1. Introduction

The cerebellum has long been known to be a key structure in the integration of descending motor command and ascending sensory feedback which account for the fluency and coordination of movements [1]. In cerebellar degenerative diseases such as spinal cerebral ataxia (SCA), the interlinked neural network is interrupted causing abnormalities in the excitation of targeted neurons which further worsen motor performance. An increasing number of associated genetic mutations have been identified in the past decade [2], of which SCA3 is the most prevalent, comprising about 1/3 of the general population [3]. At present, no known medical treatment can cure SCA.

The clinical symptoms of SCA include progressive ataxia, dysmetria, visual nystagmus, parkinsonism, muscular atrophy, spasticity, dysarthria, and hypotone [3]. Among these symptoms, hypermetria in patients with dysmetria is manifested as overshooting a predetermined target in limb movement [4–6]. Therefore, fast goal-directed movements such as in the finger-nose-finger test are used to examine limb coordination movements in these patients. In hypermetria, motor sequences are usually characterized by abnormal timing with delayed muscle activation and sudden interruptions of movements followed by exaggerated corrections [7, 8]. These aberrations in both timing and coordination are often due to inadequate control of agonist and antagonist muscles [7, 8].

Healthy adults present with a specific triphasic electromyography (EMG) pattern when executing a fast goal-directed movement [9]. In the first phase, an agonist burst (AG1) initiates and accelerates the movement toward the target. In the second phase, antagonist activation (ANT) halts the movement at the exact target. A second agonist burst (AG2) in the third phase then reduces the effect of ANT to accurately place the limb at the predetermined endpoint of movement. In SCA patients, the triphasic EMG pattern is abnormal and shows a delayed onset of ANT [4, 10–13]. Several studies have suggested that the triphasic EMG pattern is centrally programmed [14, 15] in a feedforward mode independent of any sensory feedback [14, 16], with the cerebellum involved in the regulation of cortical premovement activity [17]. Transcranial magnetic stimulation (TMS) has shown that motor evoked potentials (MEPs) of agonist muscles are facilitated around 70–100 ms before the onset of movement, which is also known as premovement facilitation [15, 18, 19]. Our recent study on the preactivation of slow and fast goal-directed wrist movements showed a delay between the peaks of premovement facilitated MEPs in the agonist and antagonist muscles and that the delay was well correlated with the time course of triphasic EMG activation [20]. Therefore, abnormal premovement facilitation may be an important mechanism underlying hypermetria. Interventions which can modulate premovement facilitation may therefore be useful in improving hypermetria.

Fast goal-directed movement training has been shown to enhance cortical excitability [21, 22]. In addition, ES (electrical stimulation) of the afferent nerve has been shown to enhance cortical excitability through plasticity-like mechanisms in healthy subjects and in SCA patients [23–26]. These findings raised the possibility that combining temporal electrical afferent nerve stimulation and voluntary movement training may enhance premovement facilitation and improve the triphasic EMG pattern of movement. We therefore designed a temporal ES-assisted fast goal-directed movement training program for patients with SCA, under the hypothesis that such a training program could improve the temporal pattern of antagonist premovement facilitation, triphasic EMG pattern, and hypermetria in individuals with SCA. To the best of our knowledge, no clinical studies have focused on the temporal control of cortical excitability, especially in the premovement phase.

## 2. Methods

**Figure 1:**
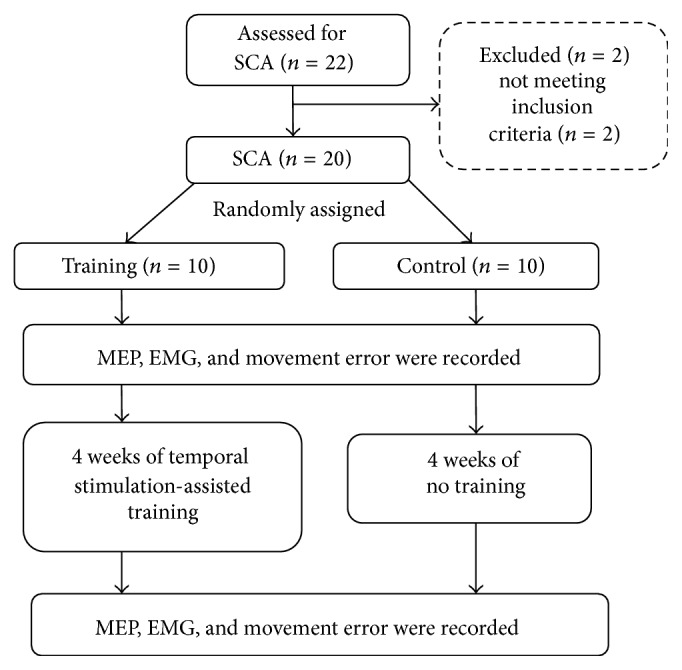
The flowchart of the study.

The study subjects were recruited from the Taiwan Spinocerebellar Ataxia Association after responding to advertisements. All of the study participants had been diagnosed with SCA (Table 1). The inclusion criteria were showing hypermetria during the finger-to-nose test, being able to sit independently to complete the experiment, no previous history of neuromusculoskeletal diseases other than SCA, and no severe tremors that would influence the recording of MEPs. Twenty-two subjects were screened, of whom 20 (age: 49 ± 8.43 years, 7 males, 13 females) met the inclusion criteria (Figure 1). The minimal sample size, which was estimated according to the data published in a previous study [20] (alpha = 0.05, power = 0.95), was 18. All of the study subjects provided informed consent, and the testing protocols were approved by our internal review board in accordance with the Helsinki Declaration. All clinical tests were performed by a licensed physical therapist who was blinded to group allocation. In addition, the subjects were also blinded to the purpose of this study.

### 2.1. Electromyography Recording

The experimental setup is shown in Figure 1. The right hand of each subject was used for the test, during which it was strapped to a custom-designed wrist goal-directed movement test and training system. The system included a laser pointer to show the wrist extension angle and a target line for 30° wrist extension. As the subjects performed the wrist movement, the laser pointer showed the movement angle in real time and provided visual feedback for the subjects. The forearm was kept neutral (0° supination) with the elbow at 80° flexion and the shoulder at 10° flexion.

The surface electromyography (EMG) of the flexor carpi radialis muscle (FCR) and the extensor carpi radialis muscle (ECR) was recorded by bipolar surface electrodes with a fixed interelectrode distance of 2 cm (B&L Engineering, Canada). The recording electrodes were located on the muscle belly of the FCR and ECR, with the direction parallel to the muscle fibers. A reference electrode was placed on the styloid process. The EMG activity was preamplified by a factor of 350 and further amplified at the mainframe amplifier (Gould Inc., Valley View, OH, USA). The raw EMG data were fed through a 60 Hz notch filter and a band-pass (10–1000 Hz) filter to eliminate environmental interference and motion artifacts. EMG activity was monitored on an oscilloscope and digitized by a 12-bit resolution analog-to-digital converter (InstruNet Model 100, Input/Output A/D System, USA) at 4000 Hz.

### 2.2. Transcranial Magnetic Stimulation

The MEPs of the FCR were elicited by the TMS (Magstim 200, Magstim Co., Dyfed, UK) using a round coil with a 9 cm outside diameter with an anticlockwise-oriented current in the coil (side A facing up) to stimulate the left motor cortex. The optimal scalp location that consistently produced the largest MEPs in the target muscle (FCR) at the lower intensity was marked, and this location was used throughout the experiment. The coil was manually maintained by a custom-designed fixation frame, and the position and orientation of the coil were kept constant throughout the experiment. The resting motor threshold was defined as the minimum TMS intensity required to elicit at least five of 10 MEPs greater than or equal to 50 *μ*V in consecutive trials in the relaxed FCR [27, 28]. The stimulation intensity for the experiment was set at 20% above the resting motor threshold.

### 2.3. Goal-Directed Movement Test

After practicing several times, the subject's right arm was trapped in a custom-designed wrist goal-directed movement test and training system to perform five fast goal-directed wrist extensions. The system included two movable segments that were placed and fixed around the wrist. An electrogoniometer (SG75, Biometrics Ltd., UK) was mounted on these two segments to record the angle during movement. A laser light beam corresponding to the movement of the hand segment was projected onto a screen to provide the subjects with real-time visual feedback. The starting and target angles (30° of wrist extension) were measured and marked and constantly displayed on the screen. The subjects were instructed to perform the wrist extension as quickly as possible and to stop the movement when the laser pointer reached the targeted line indicating 30° of wrist extension. The EMG of the ECR (the agonist muscle) and FCR (the antagonist muscle) and joint angle were recorded for further analysis. The reaction time was also recorded for the following premovement MEP test.

### 2.4. Premovement MEP Test

**Figure 2:**
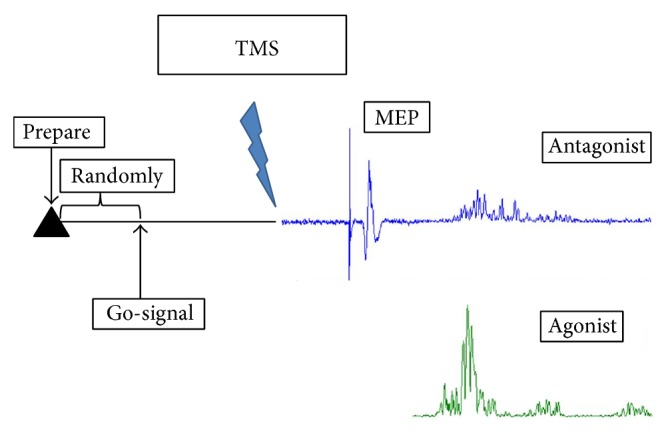
The procedure of the premovement MEP test.

An audio warning signal followed by an audio go-signal after 6–10 seconds was given through an earphone. The subjects were asked to perform the goal-directed movement test as mentioned above as soon as the go-signal was heard. The MEPs were elicited by a single pulse TMS at different time intervals in 10 ms steps after the go-signal. We wrote four sets of controlling programs, the most suitable of which were selected to assess the MEPs according to the subject's reaction time to ensure that the premovement MEPs were obtained at least 120 ms before the onset of movement (Table 2). After the assessment, the MEPs were grouped according to the onset of the agonist muscle (ECR) EMG into bins of 10, 20, 30, 40, 50, 60, 70, 80, 90, 100, and 120 ms before onset for analysis. Each of the intervals was repeated five times and delivered in a random order. Control MEPs were evoked by TMS without any audio signal (Figure 2).

### 2.5. Temporal ES-Assisted Training

After the pretest, the subjects in the training group received 4 weeks of temporal ES-assisted training at home at a training frequency of three sessions per week. During training, paired electrical stimuli were delivered every 15 seconds for 30 minutes through surface electrodes placed on the muscle bellies of the FCR and ECR. The intensity of the stimulus was set to the minimal intensity that would elicit a visible contraction of the stimulated muscle, and the pulse duration was set to 500 *μ*s. Each stimulation pair included ECR stimulation followed by FCR stimulation with an interstimulus interval of 40 ms. The 40 ms interval was chosen because our previous study on healthy subjects showed an average of a 40 ms delay between AG1 and ANT (AG1-ANT latency) in goal-directed fast movements [20]. The subjects were asked to perform the 30° fast goal-directed wrist extension movement immediately after perceiving stimulation of the ECR. The subjects in the control group received no training but some general health education. Goal-directed movement and premovement MEPs were assessed again after 4 weeks of training (training group) or in case of no training (control group), with these assessments being performed 3 days after the last training session.

### 2.6. Data Analysis and Statistics

The raw EMG data during the fast movement were transformed to the root-mean-square EMG (rmsEMG), and the onsets of ECR (AG1) and FCR (ANT) activation were calculated through rmsEMG-time curves. The onsets of ECR and FCR activation were detected when the curve passed through the threshold which was at the mean plus twice the standard deviation of the baseline. AG1-ANT latency was calculated by subtracting the ECR onset time relative to the FCR onset time and analyzed only in the trials with goal-directed movement tests without TMS or ES.

The peak-to-peak amplitude of the premovement MEPs at various time points before the onset of movement was normalized to the control MEPs. The normalized premovement MEPs were then averaged by predetermined time bins of 10, 20, 30, 40, 50, 60, 70, 80, 90, 100, and 120 ms, before the agonist muscle (ECR) EMG onset. The EMG onset of the ECR was determined for each subject in the trials without TMS in order to avoid the potential influence of TMS. The premovement MEPs elicited 130 ms before agonist muscle EMG onset were not analyzed. Linear interpolation was used to adjust the MEP amplitude if the MEPs were elicited between the aforementioned predetermined time bins.

The quality of performance was analyzed in the trials of the goal-directed movement test without TMS or ES. The final angles used to calculate the constant errors (CEs) and variable errors (VEs) were those at the end of the ballistic movement, measured before any corrective movements were made by the participant. The quality of performance was calculated using CEs and VEs. CEs, which measured the errors of goal setting, were calculated by the mean difference between the goal-directed angle and each actually performed angle (1) [29, 30]. VEs, which measured the inconsistency of repetitive measures, were calculated by the standard deviation of the difference between the goal-directed angle and each actually performed angle (2) [29, 30]. Consider

(1) $$\begin{array}{c} \text{CE}=\frac{\sum \left(Xi-T\right)}{n}, \end{array}$$

where *Xi* is the angle at which the subject stopped, *T* is the target angle, and *n* is the number of movements;

(2) $$\begin{array}{c} \text{VE}=\frac{\surd {\sum \left(Xi-M\right)}^{2}}{n}, \end{array}$$

where *Xi* is the angle at which the subject stopped, *M* is the averaged angle, and *n* is the number of movements.

Data were analyzed using SAS software version 9.1. Two-way repeated-measures analysis of variance (ANOVA) with factors of group (training and control) and time (before and 4 weeks after) followed by the post hoc Tukey test (when needed) was used to determine and compare the effect of training on premovement MEPs, VEs, and CEs. If a significant group and time interaction was found, the model was further reduced by group. The significance level was set at *P* < 0.05.

## 3. Results

There were no between-group differences in any of the measured parameters including CEs (hypermetria), AG1-ANT latencies, and MEP at baseline. The *P* values of the baseline comparisons are listed in Table 1.

### 3.1. EMG Pattern of Goal-Directed Movement Test

**Figure 3:**
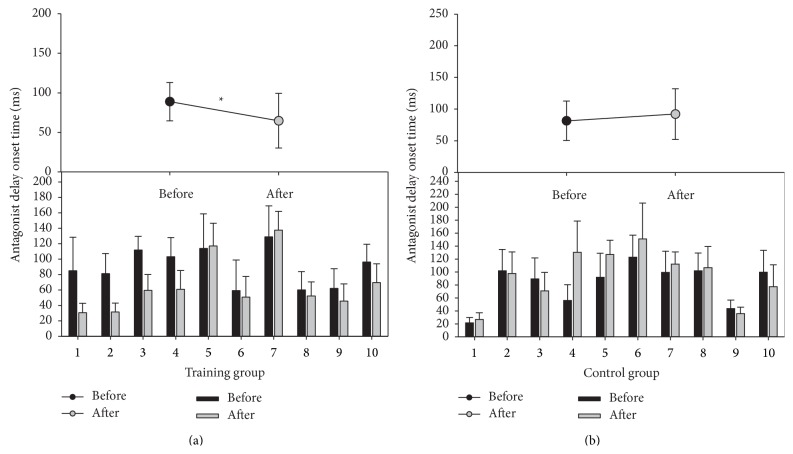
The latency of antagonist muscle activation (AG1-ANT latency) during fast goal-directed wrist extension movement in the training group (a) and the control group (b). The upper panel shows the group means and standard deviations. The lower panel shows the individual means and standard deviations. The black circle and bars indicate before training, and the gray circle and bars indicate after 4 weeks of training. *P* > 0.05 before and after 4 weeks.

ANOVA showed a significant interaction between groups and time (*F* = 8.84, *P* = 0.008). Before training, the AG1-ANT latencies were not significantly different between the training and control groups. However, after 4 weeks, the latency of antagonist muscle activation was significantly decreased to 64.66 ± 34.56 ms (*F* = 10.65, *P* = 0.0098) for the training group with no significant change in the control group (*F* = 1.37, *P* = 0.2716) (Figure 3).

### 3.2. Premovement Facilitation

**Figure 4:**
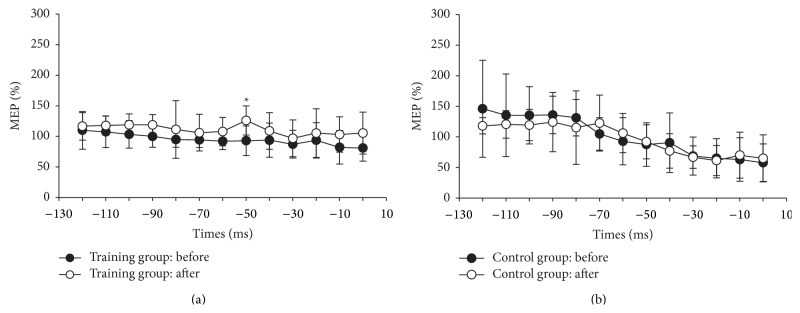
The normalized MEP amplitudes before onset latency of the antagonist muscle EMG of fast goal-directed wrist extension movement in the training group (a) and the control group (b). The black circles indicate the group mean before training, and the white circles indicate the group mean after the 4-week training program. The error bars indicate the standard deviations.

Figure 4 shows the premovement MEPs before and after 4 weeks of training in the training and control groups. Before training, the premovement MEPs of the antagonist muscle were not facilitated in either the training or control group in the fast goal-directed wrist movements. A significant group and time interaction (*F* = 5.4, *P* = 0.0336) was found 50 ms before the onset of movement (Table 3). In the training group, the premovement MEPs were significantly enhanced from 92.98 ± 24.31% to 126.16 ± 23.77% (*P* < 0.05) 50 ms before the onset of movement. However, the normalized MEPs did not change in the control group (*P* > 0.05).

### 3.3. Performance

**Figure 5:**
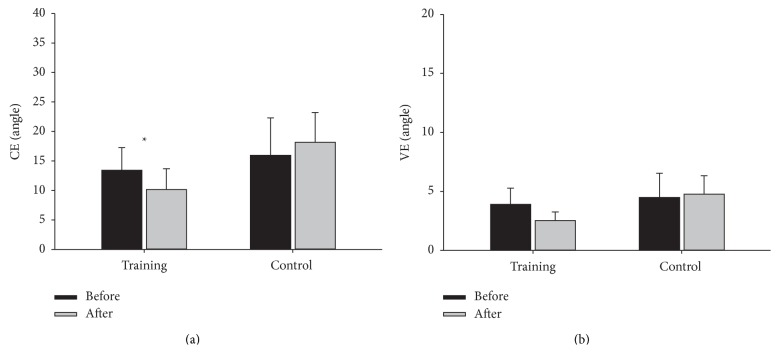
The group means and standard deviations of the CE (a) and the VE (b) of the training group (black bars) and the control group (gray bars) before and after 4 weeks.

Before training, there was no difference in CEs between the training and control group (*F* = 1.123, *P* = 0.303). Two-way ANOVA showed a significant group and time interaction (*F* = 5.99, *P* = 0.0249), with the CE decreased to 10.16 ± 3.53 degrees in the training group (*F* = 6.43, *P* = 0.0319) but unchanged in the control group (*F* = 1.48, *P* = 0.2554) (Figure 5(a)). Before training, the VEs were 3.91 ± 1.37 degrees and 4.49 ± 2.05 degrees in the training and control groups, respectively (*F* = 0.565, *P* = 0.462), compared to 2.52 ± 0.73 and 4.76 ± 1.56 degrees after 4 weeks. Two-way ANOVA showed no significant group and time interaction (*F* = 3.27, *P* = 0.0874) (Figure 5(b)).

### 3.4. Correlation Analysis

Spearman correlation coefficient analysis showed a median but significantly negative correlation between AG1-ANT latency and antagonist MEP amplitude at 50 ms before the onset of movement (*r* = −0.4066, *P* = 0.011). This suggests that the increase in antagonist premovement facilitation was correlated with the decrease in AG1-ANT latency. However, no other correlations were found between the other parameters.

## 4. Discussion

In the present study, 4 weeks of temporal ES-assisted movement training decreased CEs, indicating an alleviation of hypermetria in the patients with SCA. In addition, the prolonged AG1-ANT latency was shortened toward the normal range, suggesting that the training corrected the aberrant triphasic EMG pattern in these patients. Furthermore, the premovement facilitation of the antagonist muscle, which was previously absent in the patients, was reestablished after the 4-week temporal ES-assisted training program.

Corcos et al. applied goal-directed movement only training for 200 repetitions per day for 7 days and showed only marginal improvement in accuracy in healthy subjects [31]. In the present study, we showed for the first time that a combination of temporal ES and movement training reduced CEs, which evaluate a subject's tendency to be directionally biased when performing a skill relating to the goal setting [29, 30], indicating that the coarseness of the movements in dysmetria was improved. We suggest that the effect of the temporal ES-assisted training program was through the synergistic effect of the ES and motor training. Repeatedly pairing stimulation with ES to the peripheral nerve and TMS to the motor cortex is commonly used to induce plasticity in the brain of conscious humans [32]. Hence, pairing ES and motor training may also induce a change in plasticity in the brain to enhance the training effect.

In the present study, the patients with SCA had a prolonged AG1-ANT latency compared to the healthy controls [20]. Activation of the antagonist muscle has been reported to change fast goal-directed movements [11, 33, 34]. The delayed onset of antagonist muscle activation can explain the hypermetria symptoms in patients with SCA. After 4 weeks of training, the AG1-ANT latency had significantly decreased, which may, at least in part, explain why the CEs improved, thereby resulting in better movement control by alleviating dysmetria. In healthy subjects, only an earlier peak of antagonist muscle EMG but no similar reduction in AG1-ANT latency has been shown after movement training [31, 35]. Instead, improvements in movement errors after pure movement training have been explained by increased recruitment rates of the antagonist muscle [35]. We suggest that further studies are warranted to investigate reductions in AG1-ANT latency.

We also found that the temporal ES-assisted training program partially restored premovement facilitation towards a normal pattern [20], although the significance of the facilitation at 50 ms before activation was marginal. The underlying mechanism remains to be elucidated. It is known that movement training can enhance excitability of the movement mapping cortical area [21, 22, 36], and it is generally considered to function through a long-term potentiation-like mechanism in which the horizontal synaptic connections in the brain cortex are enhanced through motor learning [22, 36–39]. Moreover, McDonnell and Ridding reported that subjects who received 1 hour of peripheral ES to the muscle responsible for movement prior to movement training had significant improvements in motor performance, suggesting that the peripheral ES enhanced motor performance and motor learning ability [40]. Peripheral ES has also been reported to facilitate the MEPs of the corresponding innervation muscles through plasticity-like mechanisms in various patients, including those with SCA [23–26].

Previous studies have indicated that the triphasic EMG pattern is centrally programmed [15, 41–43] and stored in the motor cortex [14, 17, 44]. The correlated enhancement of premovement facilitation and the reduction of AG1-ANT latency after temporal ES-assisted training in our study may support the centrally programmed theory of triphasic EMG pattern in ballistic movements. However, we failed to show other significant correlations between measurements, and it is possible that the relationship between the physiological improvements and functional improvements is complex and nonlinear.

Although VEs were slightly decreased after training, they did not reach a statistically significant difference, in contrast to the CEs. VE measures the inconsistency of performance [29, 30], and no significant improvement in VE suggests that the temporal ES-assisted fast goal-directed movement training did not improve the variability between each trial. The potential mechanism of the training effect in the present study is that the temporal ES-assisted training helped to rebuild the central program of the triphasic EMG pattern through changes in plasticity in the neural network. The fixed temporal pattern of ES that triggered the movement improved the accuracy of the muscle contraction pattern during the requested task resulting in better CEs. In contrast, the extension range of the training movement was not strictly controlled or trained, and it is therefore not surprising to see no improvements in VEs.

In the present study, we concentrated on the modulation effect of the temporal ES-assisted fast goal-directed movement training that improved the movement in the upper limbs functionally and physiologically in patients with SCA. However, it is unclear whether ES or movement training alone can achieve a similar or different effect. Therefore, further studies are warranted to explore the effect of ES or movement training alone and in combination. Another concern may be the relatively small subjects number (10 in each group) as compared to other physiological studies [45, 46]. This limitation is due to the difficulty of recruiting patients for a long-term training study. Hence, we cannot fully exclude the possibility of the error and the accuracy problem caused by the small sample size. Moreover, only single joint movement with fixed angle, stimulus intensity, and interval was tested in the present study. Although the protocol was effective, it is not warranted to be the best.

In conclusion, 4 weeks of movement training guided by alternating temporal ES on the agonist and antagonist muscles shortened the latency between agonist and antagonist muscle activities, restored premovement facilitation, and improved movement accuracy in patients with SCA. Therefore, temporal ES-assisted fast goal-directed movement training can be considered to be a convenient and helpful therapeutic modality to improve hypermetria and may potentially be useful for patients with dysmetria caused by diseases including stroke, multiple sclerosis, multiple system atrophy type C, and other brain lesions.

## Figures and Tables

**Table 1:** Basic data of the participants.

	Groups		*P*
	Training	Control	
Number	10	10	—
Age (years)	47 ± 8	51 ± 9	0.34
Gender (F/M)	8/2	5/5	—
Onset duration (ms)	8.60 ± 6.16	10.20 ± 2.36	0.48
	Type III SCA (*n* = 4)	Type III SCA (*n* = 6)	
	Type VI SCA (*n* = 2)	Unidentified (*n* = 4)	
	Unidentified (*n* = 4)		
Finger-to-nose (times per 15 sec)	11.2 ± 2.5	10.7 ± 2.4	0.66
AG1-ANT latency (ms)	88.84 ± 24.34	81.55 ± 31.12	0.57
CE (%)	13.43 ± 3.81	15.93 ± 6.39	0.3
VE (%)	3.91 ± 1.37	4.49 ± 2.05	0.46

**Table 2:** Stimulation protocol.

Program	Reaction time of the subjects (ms)	Stimulation intervals (ms after the go-signal)
1	Less than 200	0, 10, 20, 30, 40, 50, 60, 70, 80, 90, 100, 110, 120, 130, 140, 150, 160, 170, 180, 190, 200, 210, 220

2	180–250	30, 40, 50, 60, 70, 80, 90, 100, 110, 120, 130, 140, 150, 160, 170, 180, 190, 200, 210, 220, 230, 240, 250

3	200–270	50, 60, 70, 80, 90, 100, 110, 120, 130, 140, 150, 160, 170, 180, 190, 200, 210, 220, 230, 240, 250, 260, 270

4	240–310	90, 100, 110, 120, 130, 140, 150, 160, 170, 180, 190, 200, 210, 220, 230, 240, 250, 260, 270, 280, 290, 300, 310

**Table 3:** Group means ± standard deviation of premovement MEP, AG1-ANT latency, CE, and VE.

Time	Groups				Statistical analysis	
	Training before	Training after	Control group before	Control group after	Group × time interaction	
					*F*	*P*
	MEP (% of control MEP)					

−120	110.0 ± 30.9	116.6 ± 22.5	149.0 ± 84.0	118.4 ± 14.1	1.38	0.26
−110	107.5 ± 25.9	117.8 ± 15.5	136.9 ± 72.1	123.1 ± 22.6	1.25	0.28
−100	103.4 ± 22.4	119.0 ± 17.7	134.9 ± 50.1	119.8 ± 27.1	3.87	0.07
−90	99.9 ± 17.5	118.8 ± 16.7	133.9 ± 32.2	122.5 ± 51.4	3.33	0.09
−80	94.8 ± 12.5	111.3 ± 47.2	130.8 ± 31.9	117.3 ± 63.8	2.15	0.16
−70	94.3 ± 12.5	106.0 ± 29.9	102.6 ± 27.4	122.4 ± 48.9	0.14	0.71
−60	92.1 ± 13.7	107.9 ± 23.2	91.0 ± 40.8	108.7 ± 33.1	0.02	0.89
−50	93.0 ± 24.3	126.2 ± 23.8^∗^	89.2 ± 37.6	85.8 ± 21.8	5.4	0.03^∗^
−40	93.8 ± 27.9	108.9 ± 29.9	91.5 ± 52.0	75.1 ± 29.3	2.17	0.16
−30	87.2 ± 22.7	96.8 ± 30.2	65.9 ± 31.8	67.1 ± 19.4	0.22	0.65
−20	93.6 ± 29.1	105.4 ± 39.8	61.8 ± 32.8	61.7 ± 26.4	0.33	0.57
−10	82.0 ± 27.4	103.1 ± 28.9	61.8 ± 37.8	65.2 ± 36.9	0.96	0.34
0	81.1 ± 21.6	105.4 ± 34.2	56.9 ± 32.5	62.7 ± 40.5	0.77	0.39

AG1-ANT latency (ms)	88.9 ± 24.3	64.7 ± 34.6^∗^	81.6 ± 31.1	92.2 ± 40.1	10.65	0.01^∗^

CE (%)	13.4 ± 3.8	10.2 ± 3.5^∗^	15.9 ± 6.4	18.2 ± 5.0	5.99	0.02^∗^
VE (%)	3.9 ± 1.4	2.5 ± 0.7	4.5 ± 2.1	4.8 ± 1.6	3.27	0.09

∗ is significantly different from pretraining.
